# Automatic Assessment of Intelligibility in Noise in Parkinson Disease: Validation Study

**DOI:** 10.2196/40567

**Published:** 2022-10-20

**Authors:** Gemma Moya-Galé, Stephen J Walsh, Alireza Goudarzi

**Affiliations:** 1 Department of Communication Sciences & Disorders Long Island University Brooklyn, NY United States; 2 Department of Mathematics and Statistics Utah State University Logan, UT United States; 3 Factorize Tokyo Japan

**Keywords:** automatic speech recognition, Parkinson disease, intelligibility, dysarthria, digital health, artificial intelligence

## Abstract

**Background:**

Most individuals with Parkinson disease (PD) experience a degradation in their speech intelligibility. Research on the use of automatic speech recognition (ASR) to assess intelligibility is still sparse, especially when trying to replicate communication challenges in real-life conditions (ie, noisy backgrounds). Developing technologies to automatically measure intelligibility in noise can ultimately assist patients in self-managing their voice changes due to the disease.

**Objective:**

The goal of this study was to pilot-test and validate the use of a customized web-based app to assess speech intelligibility in noise in individuals with dysarthria associated with PD.

**Methods:**

In total, 20 individuals with dysarthria associated with PD and 20 healthy controls (HCs) recorded a set of sentences using their phones. The Google Cloud ASR API was used to automatically transcribe the speakers’ sentences. An algorithm was created to embed speakers’ sentences in +6-dB signal-to-noise multitalker babble. Results from ASR performance were compared to those from 30 listeners who orthographically transcribed the same set of sentences. Data were reduced into a single event, defined as a success if the artificial intelligence (AI) system transcribed a random speaker or sentence as well or better than the average of 3 randomly chosen human listeners. These data were further analyzed by logistic regression to assess whether AI success differed by speaker group (HCs or speakers with dysarthria) or was affected by sentence length. A discriminant analysis was conducted on the human listener data and AI transcriber data independently to compare the ability of each data set to discriminate between HCs and speakers with dysarthria.

**Results:**

The data analysis indicated a 0.8 probability (95% CI 0.65-0.91) that AI performance would be as good or better than the average human listener. AI transcriber success probability was not found to be dependent on speaker group. AI transcriber success was found to decrease with sentence length, losing an estimated 0.03 probability of transcribing as well as the average human listener for each word increase in sentence length. The AI transcriber data were found to offer the same discrimination of speakers into categories (HCs and speakers with dysarthria) as the human listener data.

**Conclusions:**

ASR has the potential to assess intelligibility in noise in speakers with dysarthria associated with PD. Our results hold promise for the use of AI with this clinical population, although a full range of speech severity needs to be evaluated in future work, as well as the effect of different speaking tasks on ASR.

## Introduction

Parkinson disease (PD) is the second most common neurodegenerative disease, following Alzheimer disease [1]. Approximately 1 million individuals are estimated to be affected by the disease in the United States [2], and its prevalence surpasses 6 million people worldwide [3], with numbers projected to increase in the future [2]. Close to 90% of individuals with PD evidence problems with voice or speech, an impairment known as hypokinetic dysarthria, which has a latency that averages 7 years post–disease onset [4]. This motor speech disorder is characterized by hypophonia (ie, reduced loudness), monopitch, monoloudness, articulatory imprecision, reduced stress, short rushes of speech, and variable rate [5]. As a result, many individuals affected by the disease complain of intelligibility problems (ie, their ability to be understood by others) [6], especially in noisy environments (eg, when dining out at a restaurant). Additionally, the presence of background noise has been shown to negatively affect even speakers with mildly dysarthric speech [7]. Overall, these speech deficits substantially reduce speakers’ social participation and overall quality of life [8], as their inability to effectively communicate with others increases their frustration and social isolation.

The application of artificial intelligence (AI) in the medical field has brought promising results to enhance communication and, ultimately, quality of life [9] in a wide range of individuals. For example, voice-assisted technology, which is used in devices such as Siri or Alexa, has become increasingly more present among individuals with a neurodegenerative disease, such as those with PD [10], and has gradually been incorporated as a potential available tool for health professionals, such as speech and language pathologists [11]. The development of automatic speech recognition (ASR) technologies has substantially advanced in the past 40 years, especially given the onset of deep learning mechanisms [12]. Most crucially, the use of ASR has been shown to be effective in estimating speakers’ intelligibility deficits for different clinical populations who may present with speech impairments [13], such as those resulting from a laryngectomy [14], a cleft palate [15], or head and neck cancer [16]. Additionally, the clinical validity of ASR has also been explored in individuals with apraxia of speech and aphasia with promising results [17,18]. Project Euphonia has achieved a large-scale data set with over 1 million recordings of disordered speech, with the ultimate goal to personalize ASR models to enhance communication in individuals who experience speech and language difficulties [19,20]. Despite the great advancements that these findings represent, however, research on the application of ASR for individuals with the motor speech disorder of dysarthria has been more limited [21-23], and it has underscored the high degree of variability that characterizes dysarthric speech [13], especially with increased speech severity levels [24]. Dimauro et al [25] explored the use of ASR with 28 individuals with dysarthria associated with PD, 22 healthy older adults, and 15 healthy young controls. In their study, the speech-to-text system focused on the recognition error rates of words from different speech tasks. Although their results upheld the use of AI as a promising resource for clinical populations, it is important to note, however, that their experiment was conducted in quiet conditions, which may not reflect the real-life challenges speakers with PD face in everyday communication. More recently, Gutz et al [26] used the Google Cloud ASR API for intelligibility measurement with 52 speakers with dysarthria associated with amyotrophic lateral sclerosis and 20 healthy controls. Additionally, the authors used noise-augmented ASR to assist the AI system in discriminating between healthy speech and mildly dysarthric speech. Results from their study showed high variability and poor internal validity of machine word recognition rate, suggesting that this technology may have limited clinical applicability for this population at this time.

Our previous pilot work examined ASR performance in multitalker babble noise to measure speech intelligibility from a reading task in 5 speakers with PD and 5 healthy adults [27]. Preliminary results supported the feasibility of AI technologies to simulate real-life challenges posed by ambient noise. Our current study was aimed at expanding our previous work with speakers with dysarthria associated with PD to preliminarily validate the use of ASR in noise with this clinical population. To that end, this study reports on the development, pilot-testing, and validation of a web-based app, *Understand Me for Life* [27], to assess speech intelligibility in noise using the Google Cloud ASR API in speakers with dysarthria associated with PD. Specifically, our aims were to (1) examine how ASR compared to human transcription, the current gold standard, when determining intelligibility accuracy scores for speakers with hypokinetic dysarthria associated with PD; and (2) determine the extent to which ASR could accurately discriminate between speakers with dysarthria and healthy controls.

## Methods

### Ethics Approval

This study was approved by the Institutional Review Board at Long Island University, Brooklyn (21/01-002-Bkln).

### Speakers

In total, 20 individuals with PD (12 women and 8 men; mean age 73.3 years; age range 62-81 years) and 20 age- and sex-matched neurologically healthy adults participated in the speech recordings for this study. Individuals with PD had to meet the following inclusion criteria: (1) having a medical diagnosis of PD, (2) having experienced changes in their voice that represented a current concern, (3) having a stable anti-Parkinsonian medication, (4) passing the Montreal Cognitive Assessment [28], and (5) being a native speaker of English. Exclusion criteria included having received intensive voice-focused treatment in the past 2 years prior to the study and having received deep brain stimulation. Neurologically healthy speakers (12 women and 8 men; mean age 70.5 years; age range 59-84 years) with no history of motor speech impairments served as controls. Table 1 presents the speakers’ biographical details and clinical characteristics.

Dysarthria severity ranged from mild to moderate in these speakers and was assessed from a conversation sample by an experienced speech and language pathologist. Consensus with a second speech and language pathologist was obtained for the final dysarthria severity estimates [29].

**Table 1:** Speakers’ biographical details and clinical characteristics.

Speaker	Age (years)	Sex	YPD^a^	Dysarthria severity	Patient’s voice complaint
P1^b^	77	Female	9	Mild	Voice is softer and sounds are not as well-articulated
P2	77	Male	1	Mild-moderate	Voice is softer
P3	70	Female	6	Mild	Hoarseness
P4	72	Female	4	Mild	Less control over shaping words, changes in loudness, and occasional rapid breathing
P5	72	Female	7	Mild-moderate	Voice is much lower and softer and reduced intelligibility
P6	80	Female	8	Mild-moderate	Increased fatigue, hoarseness, and lack of clarity
P7	80	Female	8	Mild	Reduced fundamental frequency range for singing and “scratchy feeling” in throat
P8	67	Female	9	Mild-moderate	Lower pitch, hoarseness, voice is much softer, and reduced intelligibility
P9	65	Female	5	Mild	Recent coughing, softness of voice, and voice sounds rougher and softer than usual.
P10	78	Female	7	Mild	Slurring, voice is softer, and intelligibility has been affected.
P11	60	Female	8	Mild	Occasional reduction in loudness
P12	66	Male	7	Mild	Fluctuations in voice and voice is much softer
P14	73	Male	8	Mild	Occasional reduction in loudness and stuttering
P14	80	Female	7	Mild-moderate	Voice is softer
P15	73	Male	13	Mild-moderate	Voice is softer and more strained
P16	78	Male	4	Mild	Voice is softer, trouble finding words, and sometimes intelligibility is affected
P17	62	Male	13	Moderate	Voice is very soft, problems with intelligibility, and fast speaking rate
P18	81	Male	8	Mild-moderate	Voice is softer, breathiness, and have to clear throat more often
P19	80	Female	8	Mild	Voice is softer
P20	76	Male	7	Moderate	Soft voice and hoarseness
HC1^c^	68	Female	N/A^d^	N/A	N/A
HC2	71	Male	N/A	N/A	N/A
HC3	64	Female	N/A	N/A	N/A
HC4	67	Male	N/A	N/A	N/A
HC5	72	Female	N/A	N/A	N/A
HC6	77	Female	N/A	N/A	N/A
HC7	72	Male	N/A	N/A	N/A
HC8	71	Male	N/A	N/A	N/A
HC9	67	Female	N/A	N/A	N/A
HC10	78	Male	N/A	N/A	N/A
HC11	59	Female	N/A	N/A	N/A
HC12	61	Male	N/A	N/A	N/A
HC13	75	Female	N/A	N/A	N/A
HC14	66	Female	N/A	N/A	N/A
HC15	63	Female	N/A	N/A	N/A
HC16	63	Male	N/A	N/A	N/A
HC17	84	Female	N/A	N/A	N/A
HC18	84	Male	N/A	N/A	N/A
HC19	65	Female	N/A	N/A	N/A
HC20	83	Female	N/A	N/A	N/A

^a^YPD: years postdiagnosis.

^b^P: patient (speaker with dysarthria associated with Parkinson disease).

^c^HC: healthy control.

^d^N/A: not applicable.

### Speech Stimuli and Recording Procedures

A set of 100 grammatically and semantically correct sentences was created for this study. Sentences differed in length, from 5 to 9 words (eg, “Take care of my house while I am away”), and contained high frequency words in the English language (The English Lexicon Project) [30]. The data set was then divided into 4 different blocks of 25 randomized sentences each, with blocks having an equal number of sentences from each sentence length. Each speaker was randomized to 1 block of stimuli for speech recordings, so that each block was read by 10 different speakers. Recordings were self-paced and conducted in a quiet room in the speakers’ homes using a customized web-based app, *Understand Me for Life* [27], that the speakers could access from their mobile phones. The first author met with speakers over the Zoom videoconferencing platform (Zoom Video Communications) to explain the recording procedure and address any potential questions. Careful directions were provided to ensure a constant 8-cm (3.15 inches) mouth-to-microphone distance [31,32]. Given the possibility of PD-related motor impairments hindering adequate recordings (eg, tremors), care partners were recruited to assist speakers when necessary. Speakers were allowed to rerecord a sentence in cases of extraneous noise in the background. A brief familiarization phase was provided at the beginning of the recording session so that speakers could practice using the interface. Feedback from speakers was obtained for later app optimization.

For each recorded sentence, the app automatically embedded the speakers’ voice signal into +6-dB signal-to-noise multitalker babble noise [33] to provide an intelligibility score, defined as the percentage of words accurately understood by the ASR system. Automatic feedback on performance was provided at the end of the recording session and not after each sentence to avoid any potential priming effects that could influence sentence production on subsequent items [34].

### Multitalker Babble Noise

Multitalker babble is thought to be the most common type of environmental noise experienced by listeners [35], which, therefore, makes it more ecologically valid in speech perception experiments. For this study, 10-second sample recordings from National Public Radio were used. Audio files were manually checked to control for sudden changes in the speech signal (eg, increase in vocal intensity). Prolonged silences (ie, over 500 ms) were trimmed, followed by the equalization of the audio spectrum in a moving window. An equal number of male and female speakers was implemented in the creation of background noise [36]. The equalized audios were finally combined to render 10-talker babble [33].

### Listeners

In total, 30 neurologically healthy adults (25 women and 5 men; mean age 23.1 years; age range 18-31 years) participated as listeners in the study. Listeners were recruited via flyers and word of mouth across the New York City area. Inclusion criteria for participation required listeners to be native speakers of English; have no history of speech, language, or communication impairment; have no prior experience with motor speech disorders; and pass a bilateral pure-tone hearing screening at 25-dB hearing level at 500, 1000, 2000, and 4000 Hz [37]. Listeners were paid US $20 for their participation in the study.

### Human Transcription

Listeners completed the intelligibility assessment task free field (ie, without headphones) in a quiet space at the Long Island University campus, in Brooklyn, New York. The task was accessible through the *Understand Me for Life* portal on a MacBook Pro laptop (Apple Inc). Listeners maintained a distance of 85 cm from the loudspeakers (Logitech Z150), and the loudspeakers were placed 31 cm from each other. Listener-to-loudspeaker distance represented the typical distance between conversational partners [38]. The task took approximately 30-40 minutes to complete.

A brief familiarization phase was presented before the start of the experiment and contained 3 sentences produced by a neurologically healthy adult male speaker. Listeners were instructed to write down word by word what they heard and not worry about punctuation marks. Each listener was randomly assigned to 1 speaker per block, with block presentation being random across listeners. Therefore, each listener heard a total of 4 speakers and 100 sentences. Sentences were presented in multitalker babble, hence replicating the AI condition. To avoid abrupt onsets and offsets of stimuli, 400 ms of noise were inserted at the beginning of each sentence, and each sentence was followed by 50 ms of babble noise [39]. To obtain an average score for subsequent transcription accuracy calculations, each speaker was assigned to 3 listeners. None of the listeners required a break during the completion of this task.

### Data Analysis

#### Automatic Intelligibility Assessment

Automatic intelligibility assessment (AIA) was conducted using the Google Cloud ASR API, a speech-to-text AI system with documented low word error rate for individuals with healthy speech that is thought to be the best platform to handle dysarthric speech, although software performance is still dependent on speech severity, with high word error rates in cases of more severely affected speech [40].

For a given produced utterance (*S*) and the corresponding target sentence (*T*), stimuli were suitably padded with whitespace to ensure that both *S* and *T* were of equal length (*L*). Each word in *S* was codified with *w_s_* and each word in *T* with *w_t_*, where *s* and *t* were numbers from 0 to *L* – 1. Accuracy was calculated by the formula as follows:



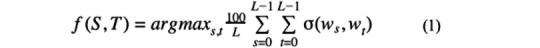



where *σ(w_s_,w_t_)* = 1 if *w_s_* = *w_t_*, and 0 otherwise. This step was implemented to avoid providing a score to words that appeared in both *S* and *T* but were out of order [27].

#### Manual Intelligibility Assessment

Transcription accuracy scores were calculated as the percentage of words correctly transcribed. Orthographic transcriptions are considered the most objective measure to assess intelligibility in dysarthria [33]. Listeners’ orthographic transcripts had to match the target to be accepted as correct [32,41]. Obvious spelling errors or errors involving homonyms did not impact calculation scores and were assessed as correct responses. Omissions or additions of morphemes (eg, flower for flowers) were coded as an error.

### Statistical Analysis

The goal of the first phase of statistical analysis was to assess the degree to which the AIA could score as well or better than the average human transcriber (ie, listener). As described above, 3 listeners orthographically transcribed sentences from the same speakers, and their data were condensed into a *percentage accuracy* measure for each sentence, which summarized the percentage of words the human listener correctly transcribed. For each question, the average percentage accuracy, denoted as *â_ij, human avg_*, was computed for each sentence *j* within each speaker *i* to reduce intralistener variability. The AIA system also received a percentage accuracy measure for each sentence or speaker, which we denoted as *â_ij, AIA_*. The success of the AIA system was defined as follows:



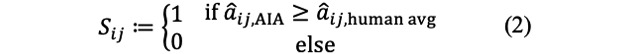



The AIA system was considered to give a successful transcription if its percentage accuracy score was at least as good as the average of the human listeners’ accuracies for sentence *j* within each speaker *i*. The data were then condensed up to the speaker level by computing the proportion of successes of the AIA system over the *j* = 1 , ... , 25 sentences read by speaker *i* as follows:



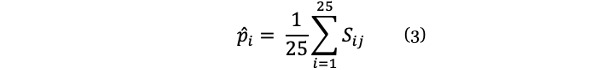



This procedure provided an estimate of the probability of success of the AIA system transcription for randomly selected speakers. Standard binomial statistics were used to quantify uncertainty in this analysis and present the results with appropriate statistical summaries and CIs. We investigated whether data provided evidence that the AIA transcriber success differed whether the system was transcribing a healthy control (HC) or a speaker with dysarthria associated with PD and whether sentence length had an effect on AIA success, via a logistic regression analysis.

The goal of the second phase of statistical analysis was to compare the ability of the resulting AIA transcription data summaries to discriminate between healthy controls and speakers with dysarthria. To investigate this goal, we applied linear discriminant analysis to identify optimal discrimination thresholds for both the listener transcriptions and the AIA transcriptions and summarized the discrimination ability of each via typical confusion matrices and correct percentage classification summaries. All statistical analyses were conducted in R statistical software (version 4.1.1; R Foundation for Statistical Computing) [42] and a discriminant and classification analysis was conducted via the *lda* function in the *MASS* package [43].

Intralistener reliability was assessed via percentage agreement on several (approximately 10) duplicate speaker sentences. Interlistener reliability was controlled for in this assessment by condensing each of the 3 listeners’ percentage accuracy measures for each speaker or sentence into the average.

## Results

**Figure 1:**
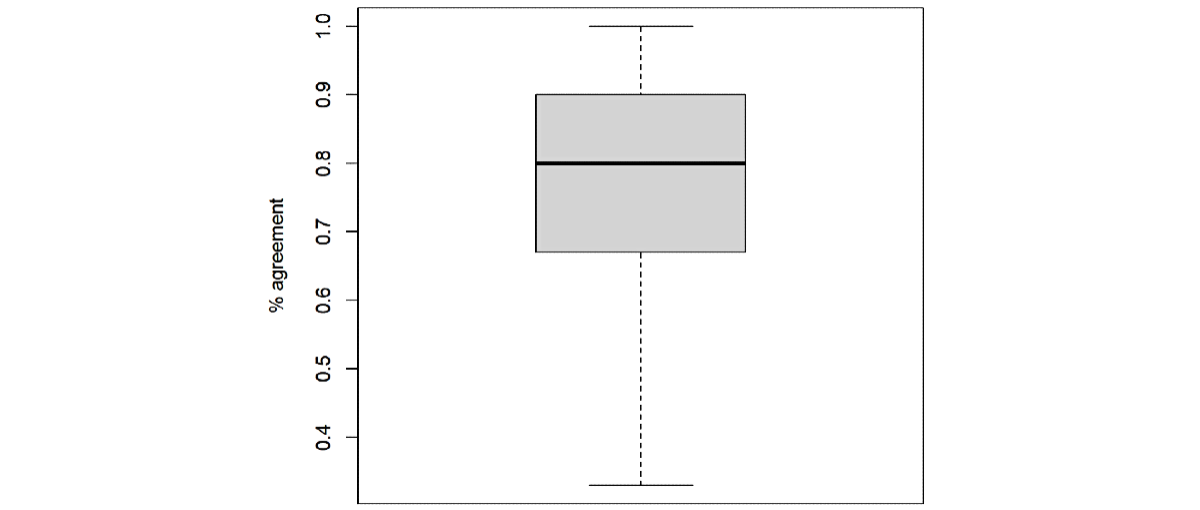
Distribution of intrarater percentage agreement across the 30 listeners.

A summary of intrarater reliability is shown in Figure 1. The average percentage agreement of repeated responses of this study’s listeners was 80%.

**Figure 2:**
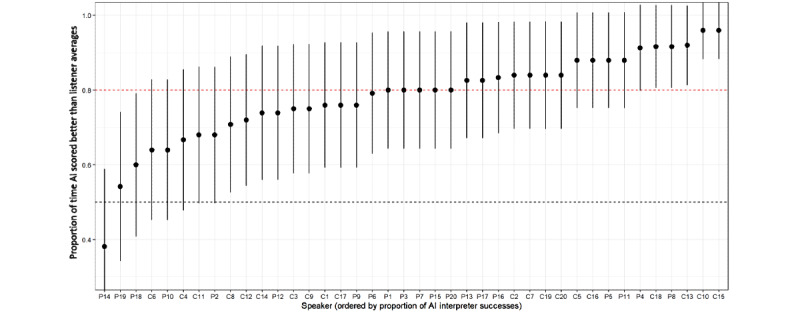
Estimates of the probability that the automatic intelligibility assessment transcriber will be as accurate as human transcribers for each speaker. The vertical bands are 95% CIs on the estimate of probability of success. Black dotted line=0.5 and red dotted line=median AI probability of success. AI: artificial intelligence; C: control; P: patient with dysarthria.

**Figure 3:**
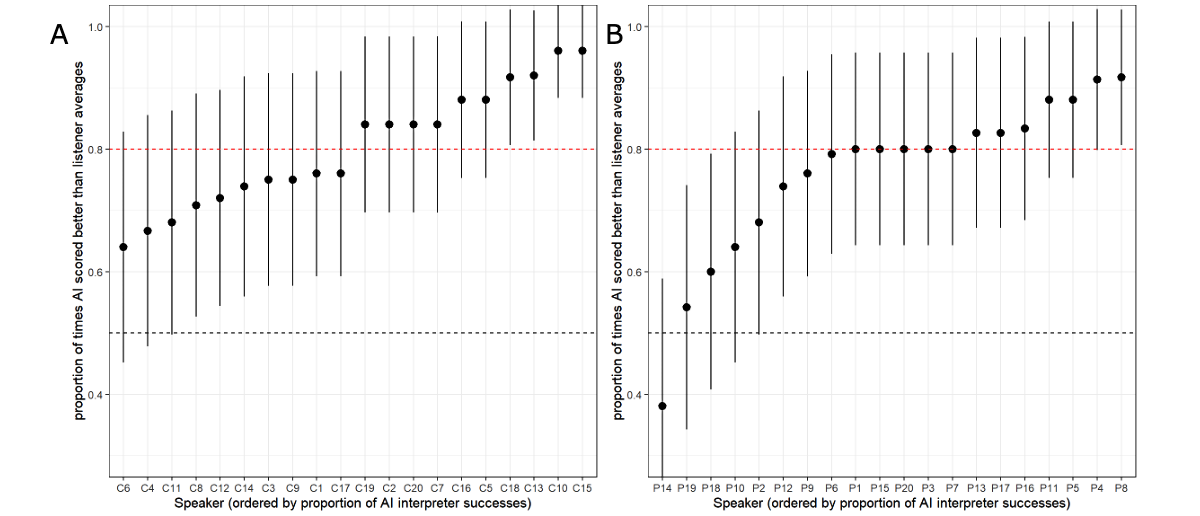
Estimates of the probability that the automatic intelligibility assessment transcriber will be as accurate as human transcribers for each speaker: (A) healthy controls and (B) speakers with dysarthria. AI: artificial intelligence; C: control; P: patient with dysarthria.

The success summaries of the AIA transcriber at the speaker level are presented in Figure 2. The figure shows estimates of the probability of success for each speaker (ordered by score) with a 95% CI. The mean probability of success is indicated by the red horizontal line. The figure illustrates that the expected success probability of the AIA transcriber for a randomly selected speaker was approximately 0.8 (95% CI 0.65-0.91), with the AIA system scoring 80% of target sentences as well or better than the human transcribers for half (22/40, 55%) of the study’s speakers. The success probability estimates stratified by speaker group (HC or speaker with dysarthria) are shown in Figure 3. The figure suggests that the AIA transcriber had a slightly more difficult time accurately transcribing the sentences read by speakers with dysarthria, with a slight decline in the estimate of probability of success for speakers #14, #18, and #19.

**Figure 4:**
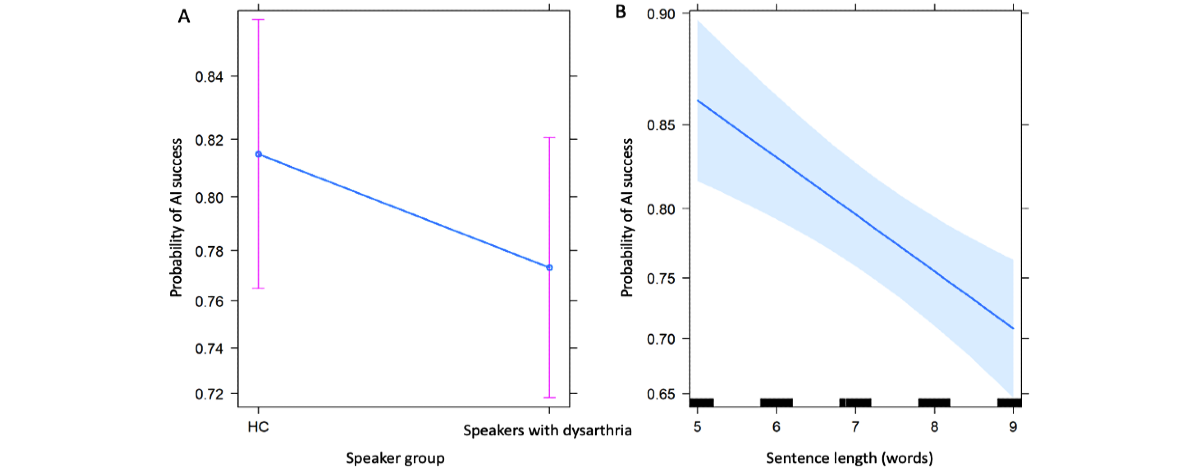
Estimated effects and CIs from the logistic regression of probability of AI success as a function of (A) speaker group, (B) sentence length, and speaker random effect. AI: artificial intelligence; HC: healthy controls.

We further analyzed these data via a logistic regression model. The response was the (logit) probability of AI success and the predictors were speaker group (HC or speakers with dysarthria) and sentence type. Speaker-to-speaker variance was controlled for by including speaker as a random effect. The fitted model estimates are presented in Table 2. The advantage of this approach is that each row provides a significance test for each term *provided we have controlled for the effects of the other terms*. In this regard, after controlling for speaker and sentence length, we see that these data provide weak evidence that AI success differs significantly by speaker group (ie, between HC and speakers with dysarthria; *P*=.23). Further, sentence length was found to have a significant negative impact on AI success (*P<*.001)*.* The results are represented in an effects plot in Figure 4. The left panel illustrates that an estimate of the probability of AI success for speakers with dysarthria is 0.78, but this value is not significantly different from the estimate of the probability of AI success for HCs (0.82; *P*=.23). The right panel illustrates an estimated dependence of the probability of AI success on sentence length, with each increase in sentence length decreasing AI success probability by an estimated 0.03.

**Figure 5:**
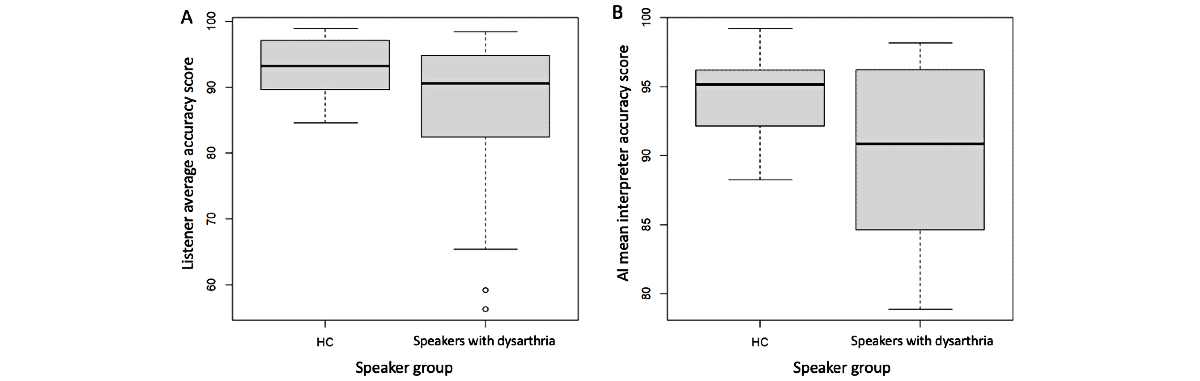
Box plots of the estimates of AIA system success by speaker category and transcriber: (A) human listener and (B) AIA system. AI: artificial intelligence; AIA: automatic intelligibility assessment; HC: healthy controls.

Percentage accuracy distributions by transcriber (human or AIA system) and speaker group are presented in Figure 5. The box plots in Figure 5 indicate that the median accuracy score for speakers with dysarthria was farther from the median accuracy score for healthy controls as compared to the distance between the 2 medians for the human transcriber data. This finding suggests that the AIA system data may offer better discrimination and classification ability for speaker group.

Confusion matrices recording the classification rates of discriminants based on human transcription data and AIA system data are presented in Table 3.

**Table 2:** Fitted logistic regression model coefficients.

Effect	Estimate	SE	*z* value	*P* value
Intercept	3.14414	0.44774	7.022	<.001
Speaker group	–0.25525	0.21156	–1.207	.23
Sentence length	–0.23658	0.05763	–4.105	.001

**Table 3:** Classification summary of the speakers based on linear discriminants fit to the human transcription data and automatic intelligibility assessment system data.

True group	Classified group via discriminant			
	Discriminant from human listener average data (overall predictive accuracy: 0.6)		Discriminant from artificial intelligence data (overall predictive accuracy: 0.675)	
	HC^a^	PD^b^	HC	PD
HC	15	5	15	5
PD	11	9	8	12

^a^HC: healthy control.

^b^PD: Parkinson disease.

## Discussion

### Principal Findings

This study aimed to develop, pilot-test, and validate the use of a web-based app, *Understand Me for Life*, to automatically measure speech intelligibility in noise in speakers with hypokinetic dysarthria associated with PD. Additionally, a secondary objective of the study was to determine whether ASR could discriminate between the speech of healthy controls and that of speakers with dysarthria.

Literature on ASR performance on clinical populations, especially those with motor speech disorders, is still sparse. To validate the use of speech-to-text technology to determine intelligibility accuracy scores for speakers with dysarthria, ASR performance was benchmarked relative to that of human transcribers [19]. Results showed that the ASR system had an 80% chance of performing as well as or better than a human transcriber on any random speaker. The potential capacity of ASR to outperform human listeners has been shown in recent studies [19], although further work is required with longer utterances and different speech tasks, as summarized in the limitations section below. Our findings also echo those reported with other clinical populations, such as those with a diagnosis of apraxia of speech and aphasia [17,18]. Additionally, our data provided no evidence that the mean probability of ASR success differed between the 2 groups of speakers, either a speaker with dysarthria or a healthy control. Thus, the success of the speech-to-text system did not depend on whether the speaker was neurologically healthy or presented with hypokinetic dysarthria associated with PD. It is important to acknowledge, however, that our speakers did not evidence dysarthria across all severity ranges; this limitation will be addressed in future work. Sentence length did influence ASR, with a decrease in accuracy observed for longer sentences, which was an expected result and is in agreement with prior literature [19,26].

The second aim of the study was to determine whether ASR could accurately discriminate between speakers with dysarthria and healthy controls. Results showed that both the human and the AIA system data provided the same classification rates for healthy controls (15/20, 75% correctly classified and 5/20, 25% incorrectly classified as speakers with dysarthria), hence evidencing equal specificity (ie, 75%). The AIA system data, however, yielded a slightly better classification success for speakers with dysarthria (12/20, 60% correct PD classifications compared to the human transcription data that only yielded 9/20, 45% correct PD classifications), which suggests stronger sensitivity than the one obtained for human transcribers (ie, 60% vs 45%). In traditional studies using human listeners, performance on intelligibility assessments has not shown significant differences between speakers with mild dysarthria secondary to PD and healthy controls [33], hence suggesting that group classification based on intelligibility scores may depend on speech severity. In our study, AI correctly classified 12 speakers with dysarthria (out of 20), a result that could be explained by the severity levels of our sample ranging from mild to mild-to-moderate only.

### Limitations and Future Work

The study’s limitations warrant future work in this research area. It should be noted that our sample of speakers with dysarthria did not include those with more severe speech deficits. Therefore, these results offer a preliminarily promising, albeit not conclusive, clinical tool for measuring intelligibility in individuals with dysarthria associated with PD. Nevertheless, ASR performance with a more diverse speech severity range in speakers with dysarthria associated with PD should be explored. It is likely that increased speech severity in individuals with PD would impact ASR, as this increase was also found in speakers with dysarthria associated with amyotrophic lateral sclerosis [26]. An additional limitation from this study is that the speech stimuli were derived from read sentences rather than from conversational speech. Although sentences rendered a higher level of predictability and, thus, control, conversational speech would have greater ecological validity. Finally, we should also acknowledge that previously reported studies used different ASR methodology compared to this study and that, as discussed in Jacks et al [18], ASR technology is in constant and rapid evolution, rendering any results on ASR in need of systematic reevaluation for the proper and valid use of ASR-assisted clinical tools.

Our ongoing work is motivated by the concept of self-management, which, in the context of a chronic illness such as PD, has become increasingly relevant. Self-management relates to the patient’s ability to identify a given behavior (eg, voice changes) and react or problem-solve in accordance with such observation [44]. Having the knowledge on how to respond to the worsening of disease symptoms and when to seek medical advice has been shown to be crucial contributors to patients’ well-being [45]. The implementation of ASR in speech intelligibility assessment, therefore, can potentially serve to establish preventative measures before the onset of speech and intelligibility degradation and control measures (eg, referral to a speech therapist) if speech deficits already exist.

### Conclusions

This study validated the use of ASR to measure intelligibility in real-life settings (ie, using background noise) in speakers with mild-to-moderate dysarthria associated with PD. Therefore, our preliminary data show that ASR has the potential to assess intelligibility in noise in this clinical population. Results hold promise for the use of AI as a future clinical tool to assist patients and speech and language therapists alike, although the full range of speech severity needs to be evaluated in future work, as well as the effect of different speaking tasks on ASR.

## Abbreviations

AI: artificial intelligence
AIA: automatic intelligibility assessment
ASR: automatic speech recognition
HC: healthy control
PD: Parkinson disease
